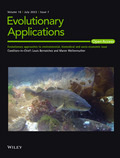# Cover Image

**DOI:** 10.1111/eva.13405

**Published:** 2023-07-21

## Abstract

Caption: Atlantic cod (Gadus morhua).

Credit: Cecilia Helmerson.